# Goodness of fit tools for dose–response meta‐analysis of binary outcomes

**DOI:** 10.1002/jrsm.1194

**Published:** 2015-12-17

**Authors:** Andrea Discacciati, Alessio Crippa, Nicola Orsini

**Affiliations:** ^1^ Unit of Nutritional Epidemiology, Institute of Environmental MedicineKarolinska InstitutetStockholmSweden; ^2^ Unit of Biostatistics, Institute of Environmental MedicineKarolinska InstitutetStockholmSweden

**Keywords:** dose‐response meta‐analysis, binary outcomes, goodness of fit, deviance, coefficient of determination, visual assessment

## Abstract

Goodness of fit evaluation should be a natural step in assessing and reporting dose–response meta‐analyses from aggregated data of binary outcomes. However, little attention has been given to this topic in the epidemiological literature, and goodness of fit is rarely, if ever, assessed in practice. We briefly review the two‐stage and one‐stage methods used to carry out dose–response meta‐analyses. We then illustrate and discuss three tools specifically aimed at testing, quantifying, and graphically evaluating the goodness of fit of dose–response meta‐analyses. These tools are the deviance, the coefficient of determination, and the decorrelated residuals‐versus‐exposure plot. Data from two published meta‐analyses are used to show how these three tools can improve the practice of quantitative synthesis of aggregated dose–response data. In fact, evaluating the degree of agreement between model predictions and empirical data can help the identification of dose–response patterns, the investigation of sources of heterogeneity, and the assessment of whether the pooled dose–response relation adequately summarizes the published results. © 2015 The Authors. *Research Synthesis Methods* published by John Wiley & Sons, Ltd.

## 1 Introduction

An important goal and challenge in epidemiologic research is to identify the shape of the association between a quantitative exposure and the risk of a binary disease. When the number of published studies reporting summarized results in terms of dose‐specific relative risks (RRs) increases, a meta‐analytical approach is necessary to synthesize the existing information on the overall shape of the dose–response relation, and to examine whether this shape is influenced by study‐level characteristics.

Because of the importance of this topic, during the last 20 years, extensive research has been carried out to develop and extend statistical methods specifically aimed at dose–response meta‐analysis of summarized data. In particular, papers investigated how to model nonlinear dose–response relations (Bagnardi *et al*., 2004; Berlin *et al*., 1993; Liu *et al*., 2009; Orsini *et al*., 2012; Rota *et al*., 2010; Takahashi *et al*., 2013), how to deal with the correlation among the RRs (Greenland and Longnecker, 1992; Hamling *et al*., 2008), how to assign typical dose values to exposure intervals (Shi and Copas, 2004; Takahashi *et al*., 2013; Takahashi and Tango, 2010), and how to evaluate the presence of publication bias (Shi and Copas, 2004). However, to the best of our knowledge, the issue of how to assess the goodness of fit of dose–response meta‐analytical models has never been specifically addressed.

In order to summarize the existing information about a certain dose–risk relation, the identification of a model that is a reasonable summary of the published dose‐specific RRs should be a natural, necessary requirement for a dose–response meta‐analysis. Therefore, data analysts should assess and report whether the posited dose–response models provide an adequate description of the data at hand. This can be performed in practice by measuring the degree of agreement between model predictions and empirical data. Although several, equally plausible dose–response models may provide an adequate fit to the data, and although a good fit alone does not necessarily mean that the “correct” model has been identified, a poor fit can raise doubts about the ability of a certain model to summarize the available data.

Despite its importance, however, goodness of fit assessment of dose–response meta‐analytical models is rarely, if ever, performed in practice. More in general, as Sutton and Higgins pointed out “[…] little formal assessment of the goodness‐of‐fit of meta‐analysis models to the data is carried out. This may be partly because many non‐statisticians conduct meta‐analysis, and to such applied researchers meta‐analysis may be seen as a necessary data‐processing procedure rather than a model‐fitting exercise” (Sutton and Higgins, 2008). We searched the PubMed database for articles published from 1 January 2015 to 31 May 2015 using the search query (“meta‐analysis” [Title] and “dose‐response” [Title]). Of the 31 identified dose–response meta‐analyses, only five of them evaluated the aforementioned degree of agreement by graphically overlaying the study‐specific RRs to the pooled dose–response relation (Fu *et al*., 2015; Liao *et al*., 2015; Sun *et al*., 2015; C. Xu *et al*., 2015; W. Xu *et al*., 2015). Although praiseworthy, this approach is misleading, as the correlation among the study‐specific RRs implies that even a well‐fitting dose–response curve might not pass through the data points.

The aim of this paper is to present and discuss three tools that, used singularly or in combination, can help to evaluate the goodness of fit of a dose–response meta‐analysis, namely deviance, coefficient of determination, and decorrelated residuals‐versus‐exposure plot. The proposed tools provide a useful framework for testing, quantifying, and graphically assessing the fit of dose–response meta‐analytical models. We show how they can be used in practice by reanalyzing data from two published meta‐analyses.

## 2 Meta‐analytic models

In this section, we briefly outline the two meta‐analytic methods employed to carry out fixed‐effects dose–response meta‐analysis of binary outcomes from aggregated data.

### 2.1 Two‐stage method

In the two‐stage approach, the meta‐analysis is carried out in two steps. In the first stage, the dose–response associations between levels of a quantitative exposure and the log(RR)s are estimated for each of the *K* studies included in the meta‐analysis (Berlin *et al*., 1993; Greenland and Longnecker, 1992). This is performed by means of the linear model

(1) $$y_{i}=X_{i}\beta_{i}+\varepsilon_{i}\text{,}$$

where *y*_*i*_ denotes the vector of log(RR) estimates for each non‐referent category against the referent one. The length of the vector *y*_*i*_, denoted by *n*_*i*_, will generally vary across studies, as indicated by the subscript *i* (*i* = 1, …, *K*).

The (*n*_*i*_ × *p*) design matrix *X*_*i*_ contains the values of the exposure for each non‐referent category, possibly including nonlinear transformations such as polynomials or splines (Bagnardi *et al*., 2004; Harrell, 2001; Orsini *et al*., 2012). For example, *p* = 1 for simple linear models (see model 1 from Example [1]), and *p* = 2 for quadratic or restricted cubic splines (RCS) models with three knots (see model 2 from Example [2]). When the exposure reference varies across studies, care must be taken to rescale the different studies' data to the same reference value (Liu *et al*., 2009).

Lastly, *β*_*i*_ is a vector of unknown regression parameters of length *p*. Because the same exposure transformations are used for all the *K* studies, both the number of columns of *X*_*i*_ and the length of *β*_*i*_ are constant across studies, as reflected by the lack of subscript *i* from *p*.

It is important to emphasize two aspects of the first‐stage models: first, the design matrix *X*_*i*_ does not include the intercept, as the log(RR) for the reference exposure value is equal to 0; second, the error terms *ε*_*i*_ cannot be assumed as independent, because the log(RR)s share a common reference group. This means that the off‐diagonal values of the covariance matrix *V*(*ε*_*i*_) = *S*_*i*_ are different from zero. Two different methods have been proposed to approximate the correlation between the non‐referent log(RR)s (Greenland and Longnecker, 1992; Hamling *et al*., 2008). For each study, the vector of regression parameters *β*_*i*_ and its variance–covariance matrix *V*(*β*_*i*_) can be efficiently estimated through the generalized least squares (GLS) estimator (Greenland and Longnecker, 1992; Orsini *et al*., 2012).

In the second stage, the study‐specific parameter estimates ${\hat{\beta }}_{i}$ are used as outcome in a multivariate fixed‐effects meta‐analysis:

(2) $${\hat{\beta }}_{i}\sim N_{p}\left(\theta ,\;V\left({\hat{\beta }}_{i}\right)\right)\text{,}$$

where *N*_*p*_ indicates a *p*‐variate normal distribution. The vector *θ* defines the pooled dose–response relation and is estimated, together with its variance–covariance matrix *V*(*θ*), using GLS (Berkey *et al*., 1998).

The second stage can be extended to multivariate meta‐regression by including study‐level covariates in Equation (2) (Gasparrini *et al*., 2012; van Houwelingen *et al*., 2002). The second stage becomes thus

(3) $${\hat{\beta }}_{i}\sim N_{p}\left(Z_{i}\theta ,\;V\left({\hat{\beta }}_{i}\right)\right)\text{,}$$

where *Z*_*i*_ is the design matrix containing study‐level covariates. Meta‐regression can be employed to identify sources of variation in study findings, and thus, it can help explaining heterogeneity in the dose–response associations across studies.

Heterogeneity in the dose–response relation across studies can be tested at the second stage by means of the Cochran Q test (Cochran, 1954), but it should be noted that this test suffers from low power, and therefore, it is of “limited use” (Hardy and Thompson, 1998). If the study‐specific dose–response associations are described by more than one parameter, that is, if *p* > 1, then the multivariate version of the Q test is to be used (Jackson *et al*., 2012; Ritz *et al*., 2008).

### 2.2 One‐stage or “pool‐first” method

An alternative to the two‐stage method is the one‐stage or “pool‐first” method (Bagnardi *et al*., 2004; Berlin *et al*., 1993; Greenland and Longnecker, 1992; Orsini *et al*., 2006). This approach is probably conceptually easier to understand and has a more straightforward notation, because it can be written as a single linear model. It is possible to show that the one‐stage and two‐stage methods are always equivalent, and not only when linear trends are fitted (Bagnardi *et al*., 2004) (see Supporting Information).

Following this approach, the study‐specific data are combined first and then one single dose–response model is fitted to the pooled data. The data are combined by concatenating the vectors *y*_*i*_ and the matrices *X*_*i*_ row‐wise, such that $y=\left(y_{1},\text{,},\ldots ,\text{,},y_{K}\right)$ and $X=\left(X_{1},\text{,},\ldots ,\text{,},X_{K}\right)$. The fixed‐effects dose–response meta‐analysis model is

(4) y=Xθ+ε,

where *V*(*ε*) = *S* is a block‐diagonal matrix with the *i*th diagonal block being *S*_*i*_. The one‐stage model can be rewritten as

(5) $$y_{ij}=\theta_{1}x_{ij1}+\ldots +\theta_{k}x_{ijp}+\varepsilon_{ij}\text{,}$$

where *j* indexes the study‐specific non‐referent exposures (*j* = 1, …, *n*_*i*_). The model coefficients *θ* and their variance–covariance matrix *V*(*θ*) are estimated using the GLS estimator. The one‐stage model is easily extended to a meta‐regression model by including interactions between exposure transformations and study‐level covariates in the design matrix *X* of Equation (4) (Berlin *et al*., 1993; Orsini *et al*., 2006).

## 3 Goodness of fit in dose–response meta‐analysis

### 3.1 Deviance

In the context of dose–response meta‐analysis, where the data points to be fitted are the non‐referent log(RR)s, the analysis of the estimated residuals $e=y-X\hat{\theta }$ is useful to evaluate how close reported and fitted log(RR)s are at each exposure level. A statistic for the absolute goodness of fit is the deviance statistic, which is defined as

(6) $$D=\left(y-X\hat{\theta }\right)\prime S^{-1}\left(y-X\hat{\theta }\right)=e\prime S^{-1}e\text{,}$$

and is a measure of the total absolute deviation between reported and predicted log(RR)s, taking into account the covariance structure of the residuals. The smaller the deviation, the closer the reported and fitted log(RR)s will be. The deviance is known, in the context of GLS estimation, as the generalized residual sum of squares (Draper and Smith, 1998).

This statistic provides a test for model specification. When the model is correctly specified, *D* is asymptotically distributed as a chi‐square random variable with *n* − *p* degrees of freedom, where *n* is the total number of non‐referent log(RR)s for all the *K* studies, that is, $n=\sum_{i=1}^{K}n_{i}$. Testing for model specification corresponds to testing whether, under the null hypothesis that the fitted model is correctly specified, the residual variance is larger than expected. A small *p*‐value calculated from this statistic is an indication that the model fails in accounting for the observed variation in the reported log(RR)s. A large *p*‐value, however, does not allow one to conclude that the model adequately explains all the observed variability.

Difference in deviances can also be used to compare the relative goodness of fit of two nested models. Suppose we have two different dose–response models, *M*_1_ and *M*_2_, where *M*_1_ is nested in *M*_2_; that is, *M*_2_ contains the parameters in *M*_1_ plus *q* additional parameters. Their deviances are *D*(*M*_1_) and *D*(*M*_2_), respectively. Under the null hypothesis that *M*_1_ provides as good a fit to the data as the more complex model *M*_2_, *D*(*M*_1_) − *D*(*M*_2_) is chi‐square distributed with *q* degrees of freedom. If the *q* additional parameters of the more complex model refer to interactions between the exposure and study‐level covariates, this test can be a useful tool for assessing heterogeneity. In particular, if the model with the interaction terms fits better the data, it is an indication that heterogeneity is present and therefore that the shape of the dose–response relation varies according to the values of the study‐level covariate.

### 3.2 Coefficient of determination R ^2^

A descriptive goodness of fit statistic that can be used as a complement to the deviance is the coefficient of determination (*R* ^2^). *R* ^2^ evaluates the degree of agreement between model predictions and empirical data and, unlike the deviance, is a standardized measure (i.e. bounded between 0 and 1) (Hagquist and Stenbeck, 1998; Kvålseth, 1985).

Derivation of the coefficient of determination for dose–response meta‐analysis follows that for GLS estimation. Given that the generalized total sum of squares is *y* ′ *S* ^− 1^ *y*, and given the lack of the intercept term (Eisenhauer, 2003; Hahn, 1977), the coefficient of determination is defined as follows (Buse, 1973; Theil, 1961):

(7) $$R^{2}=1-\frac{\text{GRSS}}{\text{GTSS}}=1-\frac{\left(y-X\hat{\theta }\right)\prime S^{-1}\left(y-X\hat{\theta }\right)}{y\prime S^{-1}y}\text{.}$$

*R* ^2^ is a dimensionless measure ranging from 0 to 1 that measures the proportion of the generalized total sum of squares accounted for by the exposure and study‐level covariates. *R* ^2^ is 0 if all the estimated coefficients in theta are 0 and therefore if the model explains no variability in the reported log(RR)s. On the other hand, *R* ^2^ is 1 if the model fits perfectly the data, which means that the model covariates account for all the observed variability among the reported log(RR)s. A low *R* ^2^ might be an indication that a different, possibly more flexible, transformation of the exposure is needed, and/or that there is large variability in the reported log(RR)s given levels of the exposure, which can be addressed through meta‐regression.

By construction, the coefficient of determination will never decrease as additional regression covariates are included in the meta‐analysis. An adjusted version of *R* ^2^ that is penalized for the number of covariates included in the model is given by

(8) $$R_{adj}^{2}=1-\frac{n}{n-p}\left(1-R^{2}\right)\text{.}$$

The adjusted coefficient of determination $R_{adj}^{2}$ increases only if the increase in *R* ^2^ is greater than what would be expected by chance alone and can be used to compare the fit of non‐nested models.

### 3.3 Visual assessment

**Figure 1:**
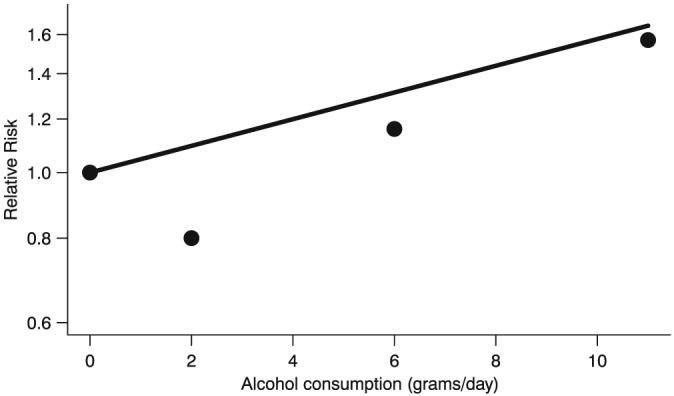
Fitted linear trend (solid line) based on Relative Risks (filled circles) reported in a single study on alcohol consumption and breast cancer risk (Greenland and Longnecker, 1992). Due to the correlation among the Relative Risks, the linear trend does not pass through the data points. The Relative Risks are plotted on the log scale.

Although deviance and *R* ^2^ are useful statistics for evaluating model adequacy, visual inspection of the model fit is strongly recommended, as it could reveal important data features and model shortcomings that would otherwise go undetected (Kvålseth, 1985). Visual examination of the goodness of fit in dose–response meta‐analysis is however complicated by the fact that the log(RR)s are correlated. This means that the fitted dose–response curve, depending on the specific correlation structure of the residuals, might not even pass through the data points. We illustrate this issue in Figure 1 using summarized data reported by Greenland and Longnecker (1992). A plot overlaying the pooled dose–response curve to the reported log(RR)s might therefore be misleading. To circumvent this problem, one can use a scatter plot where the decorrelated residuals are plotted against the exposure.

To do so, we decompose the covariance matrix *S* through Cholesky factorization, so that *S* = *CC* ′, where *C* is a lower triangular matrix. We then decorrelate the residuals by multiplying the inverse of *C* by the difference between reported and fitted log(RR)s, so that $e^{*}=C^{-1}\left(y-X\hat{\theta }\right)=C^{-1}e$. The decorrelated residuals e* are then plotted against the exposure.

The interpretation of this plot is analogous to that of the residual‐versus‐predictor plot that is used as a goodness of fit tool after classic ordinary least squares regression. Although the vertical distances from the reference line are not directly interpretable, from this plot, it is possible to evaluate how the pooled dose–response curve fits the data according to the exposure levels. If the fit is perfect, all the points will lie on the horizontal line e* = 0 (reference line). As the fit gets worse, the points will move away from the reference line. The presence of a pattern might indicate that the fit of the model is adequate only for certain levels of the exposure, suggesting the need of a more complex model. Possible extensions to this plot include overlaying a locally weighted scatterplot smoother (LOWESS) to help discerning possible patterns and, for meta‐regressions, changing the shape of the data points to distinguish them according to study‐level covariates.

### 3.4 Goodness of fit of study‐specific dose–response models

All the three tools presented so far can be equally employed to assess the goodness of fit of the study‐specific dose–response models (Equation 1). Only minor modifications in the formulas are necessary, which are briefly illustrated in the succeeding text.

The deviance for the *i*th study is defined as

(9) $${\tilde{D}}_{i}=\left(y_{i}-X_{i}{\hat{\beta }}_{i}\right)\prime S_{i}^{-1}\left(y_{i}-X_{i}{\hat{\beta }}_{i}\right)={\tilde{e}}_{i}\prime S_{i}^{-1}{\tilde{e}}_{i}\text{,}$$

and when the study‐specific model is correctly specified, it follows a chi‐square distribution with *n*_*i*_ − *p* degrees of freedom. Furthermore, because the *K* studies are assumed to be independent, it is possible to set up a joint test for model specification for all the *K* study‐specific models. In fact, under the null hypothesis that all the *K* models are correctly specified, the sum of the *K* study‐specific deviances is distributed as a chi‐square random variable with $\sum_{i=1}^{K}\left(n_{i}-p\right)=n-K\times p$ degrees of freedom, that is,

(10) $$\tilde{D}=\sum_{i=1}^{K}{\tilde{D}}_{i}=\sum_{i=1}^{K}{\tilde{e}}_{i}\prime S_{i}^{-1}{\tilde{e}}_{i}\sim \chi_{\left(n-K\times p\right)}^{2}\text{.}$$

This follows immediately from the fact that the sum of independently distributed chi‐square random variables is again a chi‐square random variable (Forbes *et al*., 2011).

The coefficient of determination for the *i*th study is defined as

$$R_{i}^{2}=1-\frac{\left(y_{i}-X_{i}{\hat{\beta }}_{i}\right)\prime S_{i}^{-1}\left(y_{i}-X_{i}{\hat{\beta }}_{i}\right)}{y_{i}\prime S_{i}^{-1}y_{i}}\text{.}$$

Lastly, the study‐specific decorrelated residuals are calculated as ${\tilde{e}}_{i}^{*}=C_{i}^{-1}\left(y_{i}-X_{i}{\hat{\beta }}_{i}\right)=C_{i}^{-1}{\tilde{e}}_{i}$, where $C_{i}^{-1}$ is the inverse of the lower triangular matrix *C*_*i*_ obtained from the Cholesky factorization of *S*_*i*_.

## 4 Examples

We will now illustrate how the deviance, the coefficient of determination, and the residuals‐versus‐exposure plot can help in evaluating and reporting the goodness of fit of dose–response models by using data from two published meta‐analyses. The selected examples are different in terms of number of studies, number of non‐referent log(RR)s, presence of nonlinearity, and/or statistical heterogeneity. We will follow the one‐stage approach to present the meta‐analytical models used in the examples. The complete R code to replicate the results is available at http://github.com/anddis/goodness‐of‐fit‐meta‐analysis.

### 4.1 Example 1: lactose intake and risk of ovarian cancer

The first example uses data from a meta‐analysis on lactose intake and risk of ovarian cancer (Larsson *et al*., 2006), including a total of 708 cases among 170 327 participants from three cohort studies and 2253 cases and 3386 controls from six case‐control studies. The analytical dataset comprised therefore nine studies, for a total of 28 non‐referent log(RR)s.

We started by fitting a linear model for the association between the log(RR)s and lactose intake (*x*_*ij*_):

(11) $$y_{ij}=\theta_{1}x_{ij}+\varepsilon_{ij}\text{.}$$

**Figure 2:**
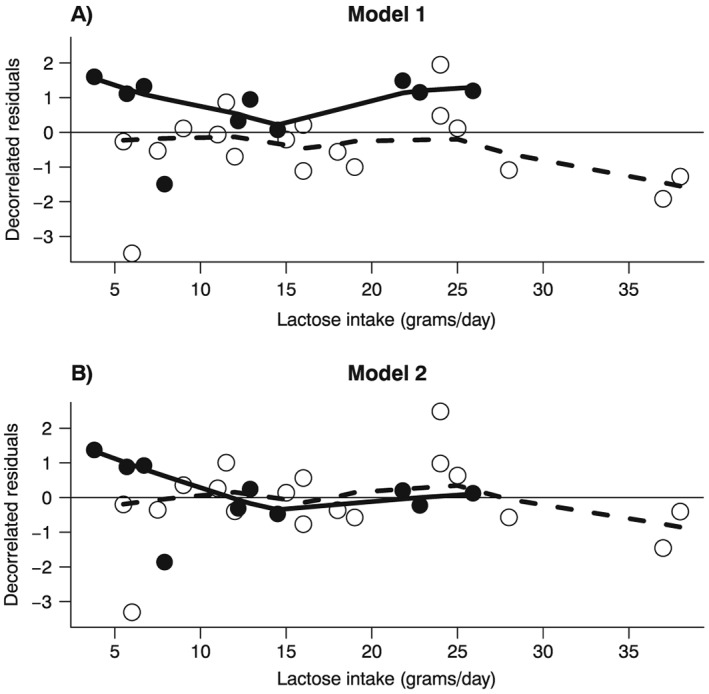
Example 1 (Larsson et al., 2006): decorrelated residuals‐versus‐exposure plots. Decorrelated residuals and LOWESS smoother for Model 1 (Panel A) and for Model 2 (Panel B). Filled circles are the decorrelated residuals of cohort studies; empty circles are the decorrelated residuals for case‐control studies. The solid line is the LOWESS smoother for decorrelated residuals of cohort studies; the dashed line is the LOWESS smoother for decorrelated residuals of case‐control studies.

This model was characterized by a particularly poor fit. In particular, the test for model specification showed evidence of lack of fit (*D* = 41, *df* = 27, *p* = 0.04), while the percentage of total variability in the log(RR) explained by model 1 was a mere 1%. Moreover, a large part of between‐study heterogeneity was left unaccounted for (*I* ^2^ = 51%) (Table 1). As an additional indication of the poor fit of model 1, the decorrelated residuals of the cohort studies were mostly above 0, while those for case‐control studies were mostly below (Figure 2, panel A). Lastly, the joint test for model specification $\tilde{D}$ did not show evidence of lack of fit ( $\tilde{D}$ = 24, *df* = 19, *p* = 0.18). This might be an indication that study‐specific linear models were indeed adequate to summarize the single dose–response associations. This result strengthened the hypothesis that a study‐level covariate, possibly study design, modified the overall dose–response association.

**Table 1:** Goodness of fit and heterogeneity measures for Example 1: lactose intake and risk of ovarian cancer (Larsson *et al*., 2006).

Model	Description	Deviance	Degrees of freedom^a^	*p*‐value^b^	*p*‐value^c^	*R* ^2^ (%)	*R* ^2^ adj. (%)	Q	Degrees of freedom^d^	*p*‐value^e^	*I* ^2^ (%)
1	Linear model	41	27	0.04	—	1	0	16	8	0.04	51
2	Linear model + interaction^f^	31	26	0.21	0.002	24	18	7	7	0.43	0

a Degrees of freedom for the deviance statistic.

b *p*‐value from test for model specification.

c *p*‐value for relative goodness of fit with respect to the model on the previous row.

d Degrees of freedom for the Q statistic.

e *p*‐value from test for heterogeneity.

f Interaction with study‐level binary variable indicating cohort studies versus case‐control studies.

We therefore employed a meta‐regression model, where we added an interaction term between lactose intake and an indicator variable for the cohort studies (*z*_*i*_):

(12) $$y_{ij}=\theta_{1}x_{ij}+\;\theta_{2}x_{ij}\times z_{i}+\varepsilon_{ij}\text{.}$$

As a result, the deviance dropped to 31 (*D* = 31, *df* = 26, *p* = 0.21) and the total amount of explained variability in the log(RR), although remained quite low, increased from 1% to 24% (Table 1). The goodness of fit increased significantly relative to model 1 (*D* = 41–31, *df* = 27–26, *p* = 0.002), indicating strong evidence of heterogeneity by study design. Consequently, heterogeneity as measured by *I* ^2^ dropped from 51% to 0%. Lastly, the residual‐versus‐exposure plot reflected the improved fit of model 2 (Figure 2, panel B). The pooled RR for every 10 g/day of lactose intake was $\exp\left({\hat{\theta }}_{1}\times 10\right)=\exp\left(-0.034\times 10\right)=0.96$ (95% confidence interval: 0.91, 1.03) for case‐control studies and $\exp\left({\hat{\theta }}_{1}\times 10+{\hat{\theta }}_{2}\times 10\times 1\right)=\text{exp}\left(\left(-0.003+0.017\right)\times 10\right)=1.15$ (95% confidence interval: 1.05, 1.25) for cohort studies. The low overall *R* ^2^ coefficient (24%) was due to the lack of association among case‐control studies. On the other hand, the *R* ^2^ among the cohort studies indicated an acceptable agreement between observed and fitted log(RR)s (*R* ^2^ = 53%).

### 4.2 Example 2: coffee consumption and risk of stroke

The second example concerns a meta‐analysis on coffee consumption and risk of stroke, including 10 003 cases and 479 689 participants from 11 cohort studies (Larsson and Orsini, 2011). A total of 52 non‐referent log(RR)s were available for the analysis.

**Figure 3:**
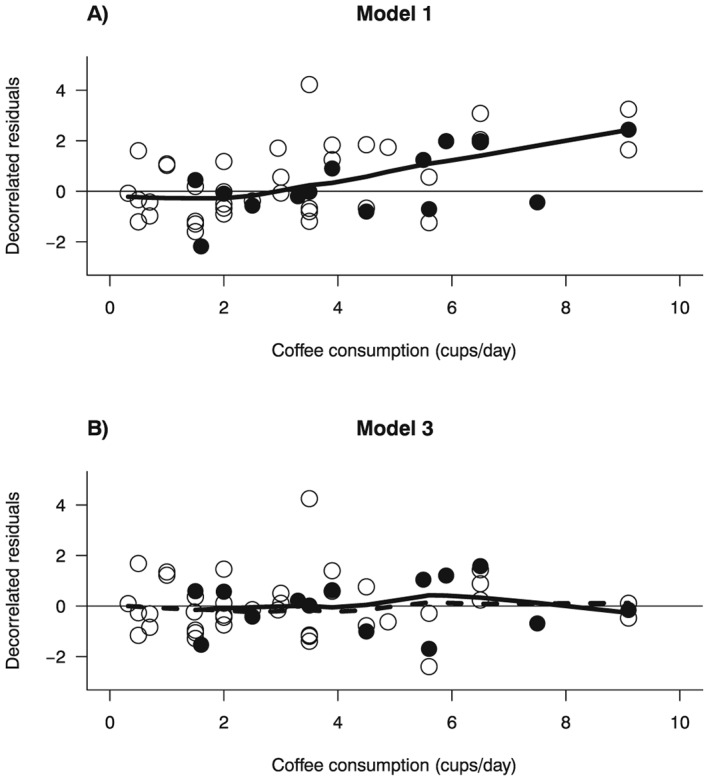
Example 2 (Larsson and Orsini, 2011): decorrelated residuals‐versus‐exposure plots. Filled circles are the decorrelated residuals of studies conducted in the Nordic countries; empty circles are the decorrelated residuals for studies conducted elsewhere. The solid line is the LOWESS smoother for decorrelated residuals of studies conducted in the Nordic countries; the dashed line is the LOWESS smoother for decorrelated residuals of studies conducted elsewhere.

We started by considering a linear model for coffee consumption (model 1), which fitted the data poorly, as indicated by a deviance of 140 on 51 *df* (*p* < 0.001) (Table 2). The residual‐versus‐exposure plot showed that the fit of the model was unsatisfactory, particularly for higher levels of coffee consumption (Figure 3, panel A). Moreover, model 1 explained only a minimal amount of between‐study heterogeneity (*I* ^2^ = 80%).

**Table 2:** Goodness of fit and heterogeneity measures for Example 2: coffee consumption and risk of stroke (Larsson and Orsini, 2011).

Model	Description	Deviance	Degrees of freedom^a^	*p*‐value^b^	*p*‐value^c^	*R* ^2^ (%)	*R* ^2^ adj. (%)	Q	Degrees of freedom^d^	*p*‐value^e^	*I* ^2^ (%)
1	Linear model	140	51	<0.001	—	41	39	76	15	<0.001	80
2	Restricted cubic spline model	75	50	0.01	<0.001	68	67	54	30	0.005	44
3	Restricted cubic spline model + interaction^f^	64	48	0.06	0.005	73	70	44	28	0.03	36

a Degrees of freedom for the deviance statistic.

b *p*‐value from test for model specification.

c *p*‐value for relative goodness of fit with respect to the model on the previous row.

d Degrees of freedom for the Q statistic.

e *p*‐value from test for heterogeneity.

f Interaction with study‐level binary variable indicating studies conducted in the Nordic countries versus studies conducted elsewhere.

To address the lack of fit of model 1, we modeled coffee consumption using RCS with three knots at fixed percentiles (25%, 50%, and 75%) of the exposure distribution:

(2) $$y_{ij}=\theta_{1}x_{ij1}+\theta_{2}x_{ij2}+\varepsilon_{ij}\text{,}$$

where *x*_*ij*1_ and *x*_*ij*2_ are the two RCS transformations of coffee consumption. The improvement in the goodness of fit of the RCS model was reflected by the increase in the *R* ^2^ coefficient from 41% to 68%, and by the large difference in the deviances between model 1 and model 2 (*D* = 140–75 = 65, *df* = 51–50, *p* < 0.001) (Table 2).

We next investigated whether a possible interaction between coffee consumption and study location could at least partly explain the statistical heterogeneity and provide a better fit of the log(RR)s. Therefore, we added to model 2 the interactions terms between the two RCS transformations and a dummy variable (*z*_*i*_) identifying the four studies conducted in the Nordic countries (Sweden and Finland):

(3) $$y_{ij}=\theta_{1}x_{ij1}+\theta_{2}x_{ij2}+\theta_{3}x_{ij1}\times \;z_{i}+\theta_{4}x_{ij2}\times \;z_{i}+\varepsilon_{ij}\text{.}$$

**Figure 4:**
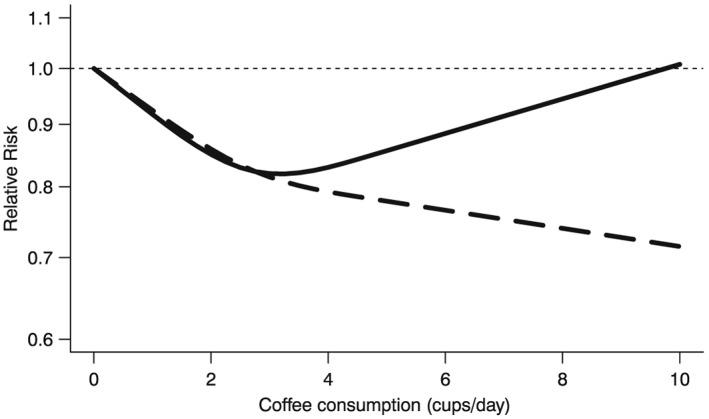
Example 2 (Larsson and Orsini, 2011): pooled dose‐response relation between coffee consumption (cups/day) and risk of stroke from Model 3 for studies conducted in the Nordic countries (dashed line) and for studies conducted elsewhere (solid line). The Relative Risks are plotted on the log scale.

The deviance decreased to 64 on 48 *df* (*p* = 0.06), while the percentage of total explained variability increased to *R* ^2^ = 73%. Even after introducing a penalty term for the two extra parameters to be estimated, model 3 fitted the data better than model 2 as indicated by the $R_{adj}^{2}$ (70% vs. 67%). Moreover, the residuals‐versus‐exposure plot no longer showed indication of lack of fit at high exposure levels (Figure 3, panel B). Finally, the test for heterogeneity of the dose–response relation between the two groups of studies was statistically significant (*D* = 75–64, *df* = 50–48, *p* = 0.005), and heterogeneity decreased to *I* ^2^ = 36% (Table 2). Using nondrinkers as the reference group, the estimated dose–response relation between coffee consumption and relative risk of stroke for the studies conducted in the Nordic countries (*z*_*i*_ = 1) was $\exp\left(\left({\hat{\theta }}_{1}+{\hat{\theta }}_{3}\right)x_{ij1}+\left({\hat{\theta }}_{2}+{\hat{\theta }}_{4}\right)x_{ij2}\right)=\exp\left(\left(-0.087+0.008\right)x_{ij1}+\left(0.077-0.037\right)x_{ij2}\right)$, while for the studies conducted elsewhere (*z*_*i*_ = 0), it was $\exp\left({\hat{\theta }}_{1}x_{ij1}+{\hat{\theta }}_{2}x_{ij2}\right)=\exp\left(-0.087x_{ij1}+0.077x_{ij2}\right)$ (Figure 4). Lastly, we calculated the RR for individuals who drank eight cups per day versus nondrinkers. The values of the first and second RCS transformation for a coffee consumption of eight cups per day were *x*_*ij*1_ = 8 and *x*_*ij*2_ = 8.2, respectively. Therefore, for the studies conducted in the Nordic countries, the estimated RR was exp(−0.079 × 8 + 0.04 × 8.2) = 0.74, while it was exp(−0.087 × 8 + 0.077 × 8.2) = 0.94 for the studies conducted elsewhere.

Overall, even though a large amount of variability in the log(RR)s was explained, there was still some evidence that the remaining variability was larger than one would expect if model 3 was indeed correctly specified (*p* = 0.06). Moreover, even after accounting for study location via meta‐regression, remaining between‐study heterogeneity was still significant (*p* = 0.03). Therefore, one might think that a better fitting model is needed.

## 5 Discussion

The main objective of this paper was to present and discuss three tools (deviance, coefficient of determination, and decorrelated residuals‐versus‐exposure plot) that can be used for testing, quantifying, and visually displaying the fit of dose–response meta‐analytical models. To the best of our knowledge, the *R* ^2^ coefficient and the decorrelated residuals‐versus‐exposure plot have never been used in the context of dose–response meta‐analysis. Furthermore, we reviewed the methods employed in the estimation of fixed‐effects dose–response meta‐analysis and showed analytically that one‐stage and two‐stage approaches are equivalent.

To illustrate how these tools can be applied in practice, we reanalyzed data from two published meta‐analyses that differed in terms of presence of nonlinearity and/or heterogeneity. These examples showed how careful scrutiny of the candidate models using the tools presented in this paper can give important indications regarding their fit.

The tools presented in this paper can be equally employed to assess the adequacy of the study‐specific models. This can be potentially useful to further examine the dose–response relation and to investigate how its shape changes across studies, thus helping to identify sources of heterogeneity. However, one limitation related to this use of the proposed tools is that the number of non‐referent log(RR) estimates reported by the single studies is generally small. As a result, the ability to assess the study‐specific models' goodness of fit is often limited.

The decorrelated residuals‐versus‐exposure plot is extendable to random‐effects dose–response models by including the covariance matrix of the random effects in the Cholesky factorization (Fitzmaurice *et al*., 2011). On the other hand, deviance and coefficient of determination *R* ^2^ are motivated via the fixed‐effects framework and lack a direct equivalent for random‐effects models. Their use for diagnostic purposes is, however, independent of the inclusion of random effects in the final model.

In conclusion, we think that the use of the goodness of fit tools presented in this paper can improve the practice of quantitative review of aggregated dose–response data. In fact, they can help the identification of dose–response patterns, the investigation of sources of heterogeneity, and the assessment of whether the pooled dose–response relation adequately summarizes the published results. By doing so, their use can yield important insights that either strengthen the conclusions drawn from a dose–response meta‐analysis, or, conversely, raise doubts about its ability to adequately summarize the available evidence.

## Supporting information

Supporting info item
