# Supplementary material for: Goodness of fit tools for dose–response meta‐analysis of binary outcomes
Source: Res Synth Methods. 2015 Dec 17;8(2):149–60. doi: 10.1002/jrsm.1194 (PMC5484373; doi:10.1002/jrsm.1194)
Supplement: Supplementary file 1 — Supporting info item [file JRSM-8-149-s001.pdf]

Goodness of fit tools for dose–response meta-analysis of binary outcomes

Supporting Information

Andrea Discacciati[1,2], Alessio Crippa[1,2], and Nicola Orsini[1,2]

[1]Unit of Nutritional Epidemiology, Institute of Environmental Medicine, Karolinska Institutet, Stockholm, Sweden
[2]Unit of Biostatistics, Institute of Environmental Medicine, Karolinska Institutet, Stockholm, Sweden

The aim of this document is to prove that one- and two-stage approaches for fixed-effects dose–response meta-analysis are equivalent.

1 Two-stage dose–response-meta analysis

In the first stage, the i-th study-specific model ($i = 1, \dots, K$) is defined as

$$y_i = X_i \beta_i + \varepsilon_i \tag{1}$$

where:

- y_i is the $(n_i \times 1)$ vector of non-referent log relative risks;
- X_i is the $(n_i \times p)$ design matrix containing the non-referent values of the dose and/or some nonlinear transformations;
- β_i is the $(p \times 1)$ vector of the i-th study-specific dose–response coefficients;
- ε_i is the $(n_i \times 1)$ vector of the error term, such that $\varepsilon_i \sim N(0, S_i)$ and S_i is considered to be known.

The study-specific vector β_i and matrix $V(\beta_i)$ are estimated using the Generalized Least Squares (GLS) estimator

$$\hat{\beta}_i = \left(X_i^\top S_i^{-1} X_i\right)^{-1} X_i^\top S_i^{-1} y_i \tag{2}$$

$$V\left(\hat{\beta}_i\right) = \left(X_i^\top S_i^{-1} X_i\right)^{-1} \tag{3}$$

In the second stage, $\hat{\beta}_i$ are pooled using a multivariate fixed-effects meta-analysis

$$\hat{\beta}_i \sim N_p\left(\theta, V\left(\hat{\beta}_i\right)\right) \tag{4}$$

The pooled estimate $\hat{\theta}$ and the corresponding (co)variance matrix $V(\theta)$ are estimated using the GLS estimator

$$\hat{\theta} = \left(\sum_{i=1}^{K} \hat{V}\left(\hat{\beta}_i\right)^{-1}\right)^{-1} \sum_{i=1}^{K} \hat{V}\left(\hat{\beta}_i\right)^{-1} \hat{\beta}_i \tag{5}$$

$$\hat{V}\left(\hat{\theta}\right) = \left(\sum_{i=1}^{K} \hat{V}\left(\hat{\beta}_i\right)^{-1}\right)^{-1} \tag{6}$$

Substituting $\hat{V}\left(\hat{\beta}_i\right)$ and $\hat{\beta}_i$ in Equations 5 and 6 with the expressions in Equations 2 and 3 gives the following estimates for $\hat{\theta}$ and $V(\theta)$

$$\hat{\theta} = \left(\sum_{i=1}^{K} X_i^\top S_i^{-1} X_i\right)^{-1} \sum_{i=1}^{K} X_i^\top S_i^{-1} y_i \tag{7}$$

$$\hat{V}\left(\hat{\theta}\right) = \left(\sum_{i=1}^{K} X_i^\top S_i^{-1} X_i\right)^{-1} \tag{8}$$

2 One-stage dose–response meta-analysis

The one-stage model is defined as

$$y = X\gamma + \varepsilon \tag{9}$$

where:

- $y = \left(y_1^\top, \ldots, y_K^\top\right)^\top$ is the $(\sum_{i=1}^K n_i = n \times 1)$ vector of concatenated study-specific non-referent log relative risks;
- $X = \left(X_1^\top, \ldots, X_K^\top\right)^\top$ is the $(\sum_{i=1}^K n_i = n \times p)$ matrix of concatenated study-specific design matrixes;
- $\varepsilon = \left(\varepsilon_1^\top, \ldots, \varepsilon_K^\top\right)^\top$ is the $(\sum_{i=1}^K n_i = n \times 1)$ vector of concatenated study-specific error terms, such that $\varepsilon \sim N(0, S)$ and

$$S = \text{diag}(S_i) = \begin{bmatrix} S_1 & \mathbf{0} & \dots & \mathbf{0} \\ \mathbf{0} & S_2 & \dots & \mathbf{0} \\ \vdots & \vdots & \ddots & \vdots \\ \mathbf{0} & \mathbf{0} & \dots & S_K \end{bmatrix} \tag{10}$$

The vector γ and the matrix $V(\gamma)$ are estimated using the GLS estimator

$$\hat{\gamma} = \left(X^\top S^{-1} X\right)^{-1} X^\top S^{-1} y \tag{11}$$

$$\hat{V}(\hat{\gamma}) = \left(X^\top S^{-1} X\right)^{-1} \tag{12}$$

Given the block-diagonal structure of S we can rewrite Equations 11 and 12 as follows

$$\hat{\gamma} = \left(X^\top S^{-1} X\right)^{-1} X^\top S^{-1} y = \tag{13}$$

$$= \left(\sum_{i=1}^K X_i^\top S_i^{-1} X_i\right)^{-1} \sum_{i=1}^K X_i^\top S_i^{-1} y_i$$

$$\hat{V}(\hat{\gamma}) = \left(X^\top S^{-1} X\right)^{-1} = \left(\sum_{i=1}^K X_i^\top S_i^{-1} X_i\right)^{-1} \tag{14}$$

3 Conclusion

Equations 7 and 8 are equal to Equations 13 and 14, respectively. This proves that $\hat{\theta} = \hat{\gamma}$ and $\hat{V}(\hat{\theta}) = \hat{V}(\hat{\gamma})$, and therefore the equivalence of one- and two-stage dose–response meta-analytical approaches.
